# Self-Reported Pain and Emotional Reactivity in Bipolar Disorder: A Prospective FACE-BD Study

**DOI:** 10.3390/jcm11030893

**Published:** 2022-02-08

**Authors:** Nathan Risch, Jonathan Dubois, Katia M’bailara, Irena Cussac, Bruno Etain, Raoul Belzeaux, Caroline Dubertret, Emmanuel Haffen, Raymund Schwan, Ludovic Samalin, Paul Roux, Mircea Polosan, Marion Leboyer, Philippe Courtet, Emilie Olié, on behalf of the FondaMental Advanced Centers of Expertise in Bipolar Disorders (FACE-BD) Collaborators

**Affiliations:** 1 Institute of Functional Genomics, University of Montpellier, CNRS, INSERM, 34094 Montpellier, France; jonathan.dubois@inserm.fr (J.D.); philippecourtet@gmail.com (P.C.); e-olie@chu-montpellier.fr (E.O.); 2 Department of Emergency Psychiatry and Post-Acute Care, CHU Montpellier, 34295 Montpellier, France; 3 Clinique de la Lironde, Clinea Psychiatrie, 34980 Saint-Clément-de-Rivière, France; 4 Fondation FondaMental, 94000 Créteil, France; katia.mbailara@u-bordeaux.fr (K.M.); irena.cussac@chpg.mc (I.C.); bruno.etain@inserm.fr (B.E.); raoul.belzeaux@ap-hm.fr (R.B.); caroline.dubertret@aphp.fr (C.D.); emmanuel.haffen@univ-fcomte.fr (E.H.); raymund.schwan@univ-lorraine.fr (R.S.); lsamalin@chu-clermontferrand.fr (L.S.); paul.roux@uvsq.fr (P.R.); MPolosan@chu-grenoble.fr (M.P.); marion.leboyer@inserm.fr (M.L.); 5 LabPsy, University of Bordeaux, EA 4139, F-33000 Bordeaux, France; 6 Department of Clinical and Academic Psychiatry, Charles-Perrens Hospital, 33076 Bordeaux, France; 7 Psychiatric Center, Hospital Princesse Grace, 1 Ave. Pasteur, 98000 Monaco, Monaco; 8 AP-HP, GHU Paris Nord, DMU Neurosciences, Hôpital Fernand Widal, 75010 Paris, France; 9 INSERM UMRS 1144-Université de Paris, 75006 Paris, France; 10 Pôle de Psychiatrie, Assistance Publique Hôpitaux de Marseille, 13005 Marseille, France; 11 INT-UMR 7289, CNRS Aix-Marseille Université, 13385 Marseille, France; 12 Department of Psychiatry, University of Paris, AP-HP, Louis Mourier Hospital, INSERM UMR 1266 Paris, 92700 Colombes, France; 13 Service de Psychiatrie de l’Adulte, CIC-1431 INSERM, CHU de Besançon, Laboratoire de Neurosciences, Université de Franche-Comté, UBFC, 25000 Besançon, France; 14 Université de Lorraine, Centre Psychothérapique de Nancy, Pôle Hospitalo-Universitaire de Psychiatrie d’Adultes du Grand Nancy, INSERM U1254, 54000 Nancy, France; 15 CHU Clermont-Ferrand, Department of Psychiatry, University of Clermont Auvergne, UMR 6602 Institut Pascal (IP), 63178 Clermont-Ferrand, France; 16 Centre Hospitalier de Versailles, Service de Psychiatrie et D’addictologie Adulte, Le Chesnay, EA 4047 HANDIReSP, UFR des Sciences de la Santé Simone Veil, Université Versailles Saint-Quentin-en-Yvelines, Versailles, France and Université Paris-Saclay, UVSQ, Inserm, CESP, Equipe “PsyDev”, 94807 Villejuif, France; 17 Université Grenoble Alpes, Inserm U1216, Grenoble Institut de Neurosciences, CHU de Grenoble, F-38000 Grenoble, France; 18 Université Paris Est Creteil (UPEC), AP-HP, Hôpitaux Universitaires «H. Mondor», DMU IMPACT, INSERM, IMRB, Translational Neuropsychiatry, Fondation FondaMental, F-94010 Creteil, France

**Keywords:** pain, depression, affective symptoms, bipolar disorder

## Abstract

In patients with bipolar disorder (BD), pain prevalence is close to 30%. It is important to determine whether pain influences BD course and to identify factors associated with pain in BD in order to guide BD management. This naturalistic, prospective study used data on 880 patients with BD from the French FACE-BD cohort who were divided into two groups according to the presence or absence of pain. Multivariate models were used to test whether pain was associated with affective states and personality traits while controlling for confounders. Then, multivariate models were used to test whether pain at baseline predicted global life functioning and depressive symptomatology at one year. At baseline, 22% of patients self-reported pain. The pain was associated with depressive symptomatology, levels of emotional reactivity in a quadratic relationship, and a composite variable of personality traits (affective lability, affective intensity, hostility/anger, and impulsivity). At one year, the pain was predictive of depression and lower global life functioning. Pain worsens mental health and well-being in patients with BD. The role of emotions, depression, and personality traits in pain has to be elucidated to better understand the high prevalence of pain in BD and to promote specific therapeutic strategies for patients experiencing pain.

## 1. Introduction

Bipolar disorder (BD) is one of the most debilitating disorders and is strongly associated with somatic comorbidities. In patients with BD, the risk of metabolic syndrome [1], cardiovascular diseases, diabetes, pneumonia, and painful conditions is increased [2]. In those patients, pain prevalence is estimated at 30%, and pain seems to influence the disease course [3]. In patients with unipolar disorders, past studies have already shown that pain impairs recovery and treatment response [4,5], increases the risk of suicide [6,7], and lowers the quality of life [8]. Conversely, in BD, only one prospective study investigated the effect of pain on life functioning, depressive, and manic symptoms [9] and found that, at one year, bodily pain was associated with manic symptoms. Therefore, the effect of pain on the prognosis of patients with BD is still unclear. Nevertheless, it is important to determine whether pain influences BD course and identify factors associated with pain in BD in order to guide BD management and develop specific therapies.

Two factors have been partially associated with pain in BD: BD subtype and depression. According to a recent meta-analysis, BD subtype II is more likely to be associated with headaches than BD subtype I [10]. Depressive symptoms have been linked to pain in many psychiatric disorders [11,12,13]. However, in BD, this association remains unclear, possibly due to a lack of statistical power and selection bias [14,15]. Previous studies concerned relatively small populations or only recruited depressed patients with BD [14]. Moreover, the association between pain and depression disappeared when analyses were adjusted for age, sex, anxiety, and perceived stress [15]. Other potential confounders should be considered when studying this link such as substance use disorders, comorbid anxiety disorders, and poor sleep quality [14,16]. For instance, poor sleep quality influences mood [17] and amplifies the experience of pain [18,19].

Personality traits have not been considered in relation to pain in BD. Nevertheless, impulsivity, hostility, affective intensity, and lability have been associated with poorer BD prognosis, such as the higher risk of suicide or substance misuse [20,21,22,23], and with pain in other clinical populations. Impulsivity has been linked to pain in patients with alcohol dependence [24], and hostility has been associated with pain in patients with chronic pain [25]. High affective lability leads to more severe pain and more functional incapacity in patients with chronic pain [26,27] and is a better predictor of pain symptoms than depression or anxiety [27].

In this naturalistic, prospective study, we assessed whether pain was associated with depression, BD subtypes, and personality traits after controlling for sleep quality, somatic and psychiatric comorbidities, medication intake, sociodemographic variables, and anxiety. We also investigated whether pain could be linked to five domains of BD functioning: emotional reactivity, cognitive processing speed, motivation levels, motor activity, and sensory perception intensity [28]. These dimensions appear relevant when evaluating pain because it has two core components: a sensory–discriminative dimension and a cognitive–affective dimension [29]. Finally, we tested whether pain at baseline predicted global functioning and depression level at one year.

## 2. Materials and Methods

### 2.1. Study Population

FACE-BD is a naturalistic, prospective cohort of French outpatients with BD enrolled at the 12 advanced Centers of Expertise in Bipolar Disorder (CEBD) and coordinated by the FondaMental Foundation. The methodology has already been described elsewhere [30,31]. Participants had a diagnosis of BD type I, II, or not otherwise specified, according to the Structured Clinical Interview for DSM-IV Axis I Disorders (SCID-I), and were older than 18 years. In total, 880 patients with BD were selected for the transversal analysis. A subsample of 368 (41.8%) patients, with a follow-up visit at 1 year, was selected for the longitudinal analysis.

### 2.2. Assessments

Sociodemographic variables (age, sex, marital status, education) and current psychotropic medication were recorded. Age at BD onset, number of thymic episodes, number of lifetime suicide attempts, somatic and psychiatric comorbidities were recorded by trained psychiatrists or psychologists, using the SCID-I. Quality of sleep was self-evaluated by the patients with the Pittsburgh Sleep Quality Index (PSQI). The total score ranges from 0 to 21, with higher scores indicating poorer sleep quality [32,33]. Global life functioning was assessed with the Functioning Assessment Short Test (FAST) [34] that includes autonomy, occupational functioning, cognitive functioning, financial issues, interpersonal relationships, and leisure time subscores. A higher total score indicates poorer functioning.

#### 2.2.1. Pain

The level of pain was self-evaluated with the EQ-5D-5L [35] questionnaire. The EQ-5D is a standardized quality of life scale, developed by the European EuroQol group. This questionnaire has been validated in several countries, including France. It includes five dimensions: mobility, self-care, usual activities, pain/discomfort, and anxiety/depression. Each dimension is rated on a 5-point Likert scale: no problem, slight problems, moderate problems, severe problems, and extreme problems. The standard reference period for the response is the respondent’s “own health state today”. 

Patients were classified into two groups according to the presence or not of moderate or severe problems for the pain/discomfort dimension [13]. The EQ-5D-5L pain dimension has good psychometric properties and has shown good responsiveness and discriminative validity in various diseases where the pain is a major symptom [36,37,38,39,40]. It is correlated to the scores of specific pain measuring tools, such as pain visual analog scales and the Brief Pain Inventory [40,41].

#### 2.2.2. Affective States

The manic state was assessed with the Young Mania Rating Scale (YMRS). The Quick Inventory of Depressive Symptoms (QIDS) scale, without the item on suicidal ideation, was used to assess the depression level. Suicidal ideation was self-evaluated with item 12 of the QIDS, as conducted in previous studies [42]. The anxious state was self-evaluated with the Spielberg Anxiety Inventory (STAI). Subscales of the Multidimensional Assessment of Thymic States (MAThyS) for patients with BD were used to evaluate emotional reactivity, cognitive processing speed, motivation level, psychomotor activity, and sensory perception. This scale has 20 items, each rated using a visual analog scale that ranges from 0 (inhibition) to 10 (hyperactivation), with 5 representing the baseline activity [28]. For each subscale, inhibition refers to low emotional reactivity, decreased motivation, slower cognition, psychomotor retardation, and attenuated sensory perceptions, whereas activation refers to emotional hyper-reactivity, increased motivation, thought acceleration, psychomotor activation, and increased sensory perceptions.

#### 2.2.3. Personality Traits

Personality traits were self-assessed with the Affective Lability Scale (ALS), the Affect Intensity Measure (AIM), the Barrat Impulsiveness Scale (BIS 10), and the Buss–Durkee Hostility Inventory (BDHI). Two dimensions—expressive and attitudinal aggressiveness—were derived from the BDHI because its construction produces these two distinct loaded factors [43,44].

### 2.3. Ethical Concerns

A web-based application, e-bipolar©, was developed and used to collect data for clinical monitoring and research purpose [31]. Access to this web-based system is carefully regulated, and this application was approved by the French body overseeing the safety of computerized databases (i.e., Commission Nationale de l’Informatique et des Libertés, CNIL) [31]. The study was performed according to the Declaration of Helsinki. The protocol was approved by an ethics committee (CPP-Ile de France IX).

### 2.4. Statistical Analysis

The normal distribution of variables was evaluated. The Box–Cox transformation was used for the QIDS (total score without the suicidal item 12) and FAST scores. When the variable was used as the outcome (FAST score), we transformed it to match the model assumption (i.e., normality). When the variable was used as a factor (QIDS), we transformed it to reduce the influence of positive skewness and the influence of outliers. The MRS and QIDS item 12 scores were categorized into three and two classes, respectively (Table 1). Variables in the two patients’ groups (with and without pain at baseline) were compared by univariate analysis. For quantitative variables, mean and standard deviation (SD) were used. For qualitative variables, the number of occurrences and frequencies per class were used. Quantitative and qualitative variables were compared between groups with the *t*-test or Mann–Whitney test, and the Chi2 or Fisher test, respectively. All analyses were performed with R 4.0.0 [45].

#### 2.4.1. Transversal Analysis

To test whether pain was associated with affective states, BD subtype, and personality traits, three multivariate models were built. For each model, confounders were selected from the literature and from univariate analysis (*p* < 0.15). Model 1 included all variables related to affective states (depression, mania, anxiety, suicidal ideation, MAThyS subscales) while controlling for confounders. Model 2 included the BD type while controlling for affective states and for the same confounders kept in Model 1. Model 3 included all variables related to personality traits while controlling for the same confounders as in Model 1 and 2. To prevent collinearity in the multivariate analysis, principal component analysis (PCA) was used to cluster highly correlated personality traits [46]. 

For each model, the relationships between covariates and outcomes were estimated using a generalized additive model (GAM) fit by penalized likelihood maximization with binomial family and logit link functions [47]. GAM has more flexibility than generalized linear models because the relationships between independent and dependent variables could be linear or nonlinear. As our model contained nonlinear effects, we used a GAM to provide a regularized and interpretable solution [48]. Nonlinearity was considered using cubic regression spline with leave-one-out cross-validation to prevent overfitting problems. To select the best model, a backward selection was performed and only the variables that gave the best fit, according to the Akaike information criterion (AIC), were retained. The odds ratio (OR) and their 95% confidence intervals (CI) were estimated. 

#### 2.4.2. Longitudinal Analysis

To test whether pain at baseline predicted global functioning and depression at one year, two models were built. Both models included pain at baseline and were adjusted for baseline sociodemographic variables, sleep quality, and depressive symptoms. Patients were considered depressed if they had a QIDS score > 5 [49]. The relationships between covariates and outcomes were estimated using a multivariate linear regression for Model 1 and multivariate logistic regression for Model 2. To select the best model, a backward selection was performed, and only the variables that provided the best fit, according to the AIC, were retained. The adjusted coefficient/OR and 95% CI were estimated to quantify the risk of greater disability and of depression according to the presence/absence of pain.

## 3. Results

### 3.1. Sample Description

At baseline, the study sample (*n* = 880) included 532 (60%) women, and the mean age was 40.31 years (SD = 12.64). Moreover, 409 (46%) patients had BD type 1, and 195 (22%) patients reported moderate-to-severe pain (EQ-5D-5L score). The mean QIDS score was 9.6 ± 5.8, and the mean YMRS score was 2.3 ± 3.7. The sample characteristics are summarized in Table 1. 

At one year, the study sample (*n* = 368) included 222 women (60%), and the mean age was 42.46 years (SD = 12.97). In the longitudinal sample, 179 (49%) patients had BD type 1, and 86 (23%) patients reported moderate-to-severe pain (EQ-5D-5L score).

Patients lost during the follow-up were younger, more educated, younger at BD onset, and took fewer medications. Conversely, the affective state was similar between patient lost and not lost to follow-up (Supplementary Material: Table S1).

### 3.2. Transversal Analysis

#### 3.2.1. Model 1: Affective States and Self-Reported Pain

**Figure 1:**
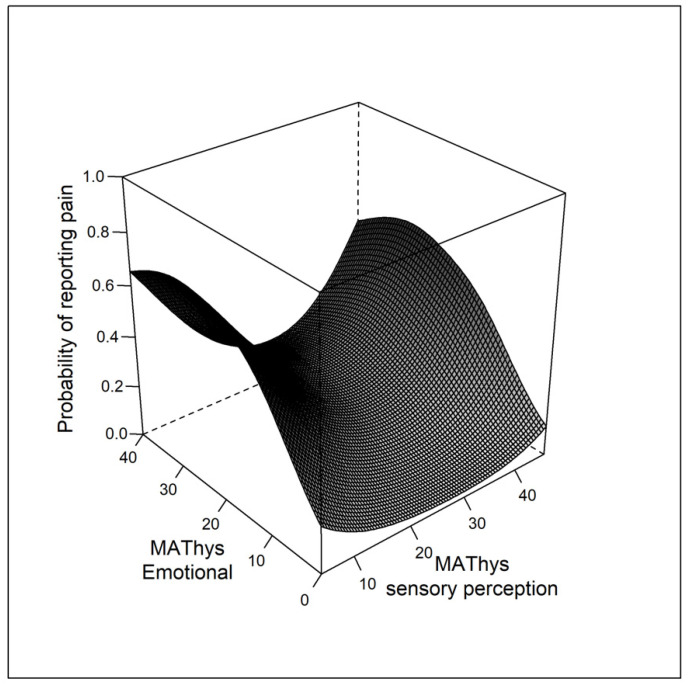
Probability of reporting pain in function of the emotional reactivity and sensory perception levels. MAThys: Multidimensional Assessment of Thymic States.

The variables excluded from the complete model according to the AIC were lifetime anxious disorder, suicidal ideation, (hypo)manic symptoms, anxiety state, and all sociodemographic variables except age and education. In the best-fitted model, self-reported pain was significantly associated with depressive symptoms (OR = 1.19 (1.09–1.30)), quality of sleep (OR = 1.10 (1.04–1.15)), and age (OR = 1.01 (1.00–1.03)) (Table 2). The MAThyS sensory and the emotional subscores were associated with pain in a quadratic relationship, with a U-shaped curve between the sensory component and pain. The probability of reporting pain was higher for patients with sensory inhibition or sensitization, compared with patients with normal sensory perceptions (*p* = 0.002) (Figure 1). The probability of reporting pain was lower in patients with emotional inhibition, compared with normal or elevated emotional reactivity (*p* = 0.008) (Figure 1). The probability of reporting pain increased with the emotional reactivity subscore until a plateau was reached (Figure 1).

#### 3.2.2. Model 2: BD Subtype and Self-Reported Pain

BD subtype II was not more likely to be associated with pain than BD subtype I (OR = 1.11 (0.76–1.62)) when age, education, level of depression, emotional and sensory subcomponents of the MAThyS subscores were considered in the model (Table 2).

#### 3.2.3. Model 3: Personality Traits and Self-Reported Pain

##### Step 1: Variable Clustering by PCA

**Figure 2:**
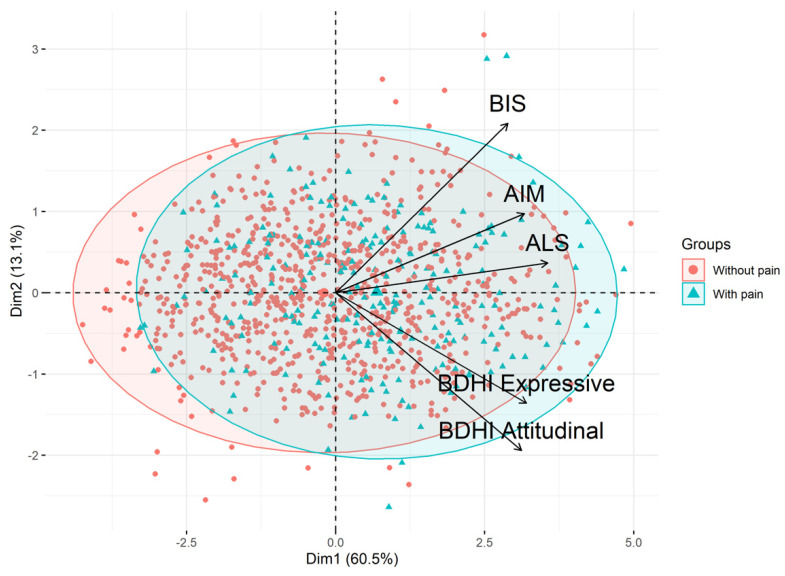
PCA clustering of personality traits evaluated with the indicated questionnaires. AIM Affect Intensity Measure; ALS Affective Lability Scale; BDHI Buss–Durkee Hostility Inventory; BIS-10 Barratt Impulsiveness Scale.

All personality trait data were summarized by PCA (Figure 2). The first PCA component explained 60.5% of the variance. Eigenvalue and percentage of variance explained by each dimension of the PCA are presented in Table S2 (Supplementary Material: Table S2). All personality trait questionnaire scores were highly correlated with the first PCA component (Table 3). The coordinate on the first component was then used to create a new variable called “borderline personality traits” that combined high affective lability, affective intensity, hostility/anger, and impulsivity.

##### Step 2: Association between Pain and “Borderline Personality Traits”

The “borderline personality traits” variable was included in a multivariate analysis adjusted for confounders (age, education, depression level, and sleep quality). In the best-fitted model, self-reported pain was significantly associated with “borderline personality traits” (OR = 1.13 (1.00–1.29)), depression (OR =1.14 (1.04–1.25)), quality of sleep (OR = 1.08 (1.03–1.14)), and age (OR = 1.02 (1.00–1.03)) (Table 2). Self-reported pain was still associated with the sensory (*p* = 0.003) and emotional (*p* = 0.023) subscores of the MAThyS.

### 3.3. Longitudinal Analysis

#### 3.3.1. Model 1: Pain at Baseline Is Associated with Global Functioning at One Year

The variables excluded from the complete model according to the AIC were age, education, and marital status. In the best-fitted model, global life functioning at one year was significantly associated with pain (ß = 1.11 (0.22–2.01)), global life functioning score (ß = 0.59 (0.47–0.71)), and quality of sleep (ß = 0.15 (0.03–0.26)). Depression (ß = −0.14 (−0.33–0.05)) and sex (ß = 0.65 (−0.09–1.38)) were kept in the model, although they were not significantly associated with global life functioning level at one year.

#### 3.3.2. Model 2: Pain at Baseline Is Associated with Depression at One Year

On the basis of a QIDS score of >5, two hundred patients (54% of 368) were classified as depressed. The variables excluded from the complete model according to the AIC were age, marital status, and quality of sleep. In the best-fitted model, depression at one year was significantly associated with pain (OR = 1.87 (1.07–3.35)), depression score (OR = 1.32 (1.21–1.45)), and being a woman (OR = 2.23 (1.40–3.57)). Education (OR = 0.64 (0.40–1.02)) was kept in the model, although it was not significantly associated with the presence of depression at one year.

## 4. Discussion

Our main results show that patients with BD and pain are at risk of depression and functional disability with poorer autonomy, independently of the baseline depression level and sleep quality. The contributing factors of pain in BD are depression level, borderline personality traits, sensory perceptions, and emotional reactivity, independent of sleep quality and somatic and psychiatric comorbidities.

Depression has been associated with pain in patients with a major depressive disorder [50], schizophrenia [13], and borderline personality disorder [12]. Our results extend this association to patients with BD, thus underlining the importance of depression on pain. One hypothesis is that patients with depression display increased excitability of nociceptive neurons (i.e., greater temporal summation) [51,52], which could lead to a higher risk of chronic pain [53].

In our study, BD subtype II was not associated with pain. This suggests that broader conditions of pain are not specifically related to BD subtypes, unlike headache. Alternatively, this result could be explained by the inclusion of depression levels in our model. As shown by our results, the trend (univariate analysis) completely disappeared when the patients’ depressed status was considered. In future studies on headache and BD subtypes, it would be interesting to assess whether the BD subtype II still has a direct effect when the depression level is considered in the statistical model.

Impulsivity, hostility/anger, affective lability and affect intensity were sufficiently collinear to be summarized by one single personality trait component that is reminiscent of borderline personality disorder. These borderline personality traits were associated with pain in BD. Pain prevalence is high also in patients with borderline personality disorder [12], and features of borderline personality disorder have already been associated with pain [25] even after controlling for depression [54]. As BD and borderline personality disorder are highly comorbid [55], the presence of borderline features in BD could explain the high prevalence of pain in these patients. Interestingly, similarly to patients with unipolar depression, the temporal summation is higher in individuals with borderline features than in controls, and this has been correlated with dysregulated affectivity [56].

Finally, the exploratory dimensional analysis revealed that the MAThyS sensory and emotional components were linked to pain in a quadratic relationship. The probability of experiencing pain was related to the impaired intensity of sensory perceptions (lower or greater) (Figure 1). Pain is a multisensorial perception in which all senses influence the integration of nociception [57,58,59,60,61]. Patients with BD often report modifications of sensory perception. In these patients, multisensorial integration could be impaired, leading to a greater risk of pain. On the other hand, for the emotional component, the probability of having pain was lower only in patients with increased inhibition (apathetic). Notably, Kraepelin and Bleuler already suggested that affective flattening partially explains pain insensitivity [62]. Conversely, patients who reported emotional hyper-reactivity and normal reactivity were more prone to report pain. This is consistent with experimental research showing that negative emotions generally increase pain intensity, and this effect depends on the level of arousal [63]. Specifically, some studies found that nociceptive neurons in the spinal cord are more excitable during unpleasant stimuli [64].

Similar to previous studies, the results revealed that pain prevalence was high in patients with BD [3]. Indeed, 22% of them reported moderate-to-severe pain at baseline, and 23% at one year. Our longitudinal analysis showed that pain affects BD course, thus underlining the large impact of pain on depression and functioning. Patients with BD are, at the same time, more likely to experience various pain conditions that can worsen their mental health and well-being, and less likely to receive adequate pain management [65,66,67]. Thus, pain assessment and management are crucial in clinical practice, and psychiatrists have a pivotal role in helping patients with BD who experience pain [68]. It has been already suggested that improving the management of pain in patients with severe mental illness might contribute to enhancing the treatment results [5]. To this aim, psychiatrists could reassess psychotropic medications and use non-pharmacological therapy for the management of pain and mood disorders. For instance, cognitive-behavioral therapy has shown good efficacy on pain, BD, and quality of sleep [69,70,71]. Mindfulness also could be interesting to improve emotion regulation in patients with BD and thus their pain [72,73].

Some limitations must be highlighted. First, the EQ-5D-5L questionnaire is a validated measure of pain intensity but does not provide any useful information on pain location, duration, and frequency. Yet, such information is important when studying pain. Second, we only measured dimensions that are reminiscent of borderline personality traits but did not assess borderline personality disorder to extend our findings. Third, there was a selection bias due to the recruitment of patients within the CEBD network. Fourth, patients were not manic at enrollment, and therefore, this study was not properly designed to test whether manic symptoms are related to pain.

Our study also has some strengths—namely, large sample size, combined dimensional and categorical measures, and adjustment for sleep, medications, somatic and psychiatric comorbidities. For instance, antidepressants and anticonvulsants are effective on pain, but in our study, they were inversely related to pain. Medication intake was proportional to the pain level. The lack of effect of such medications has already been reported elsewhere [74,75,76].

## 5. Conclusions

Our study shows that pain worsens mental health and well-being in patients with BD. This is of concern because the prevalence of pain is high and seems to be stable across time. Interestingly, all contributing factors of pain (depression level, borderline personality traits, and emotional reactivity) found in this observational study could lead to an increase in temporal summation. This link should be studied in future experimental and observational studies.

## Figures and Tables

**Table 1:** Sociodemographic and clinical characteristics of patients with and without pain at baseline.

Variable		Without Pain Mean (sd)/Number (%)	With Pain Mean (sd)/Number (%)	*p*-Value
Sociodemographic				
n		685	195	
Age (years)		39.88 (12.83)	41.83 (11.86)	0.06
Sex	Men	278 (40.6)	70 (35.9)	0.27
	Women	407 (59.4)	125 (64.1)	
Single	No	350 (51.1)	103 (52.8)	0.73
	Yes	335 (48.9)	92 (47.2)	
Education (High school diploma)	No	245 (35.8)	85 (43.6)	0.06
	Yes	440 (64.2)	110 (56.4)	
Clinical				
BD subtype	I	329 (48)	80 (41)	0.19
	II	286 (41.8)	95 (48.7)	
	NOS	70 (10.2)	20 (10.3)	
Age at BD onset (years)		23.48 (9.13)	23.89 (9.44)	0.6
Number of depressive episodes		4.96 (4.48)	6.50 (5.71)	0.0004
Number of manic episodes		1.09 (1.99)	1.00 (2.52)	0.6
Number of hypomanic episodes		3.32 (4.83)	4.10 (5.44)	0.1
Lifetime history of suicide attempt	No	463 (67.6)	121 (62.1)	0.17
	Yes	222 (32.4)	74 (37.9)	
Current substance use disorder	No	612 (89.3)	171 (87.7)	0.6
	Yes	73 (10.7)	24 (12.3)	
Lifetime anxiety disorder	No	435 (63.5)	96 (49.2)	0.0004
	Yes	250 (36.5)	99 (50.8)	
Lifetime eating disorder	No	560 (81.8)	150 (76.9)	0.16
	Yes	125 (18.2)	45 (23.1)	
Multiple sclerosis	No	661 (99.5)	188 (99.5)	1
	Yes	3 (0.5)	1 (0.5)	
Cancer	No	628 (97.2)	175 (96.7)	0.9
	Yes	18 (2.8)	6 (3.3)	
Inflammatory bowel disease	No	659 (99.2)	184(98.9)	0.65
	Yes	5 (0.8)	2 (1.1)	
Rheumatoid arthritis	No	670 (99.9)	191 (100)	1
	Yes	1 (0.1)	0 (0)	
Ulcer	No	644 (97.3)	176 (94.6)	0.12
	Yes	18 (2.7)	10 (5.4)	
QIDS-SR (without item 12) Box–Cox Transformed		5.13 (2.61)	6.82 (2.54)	<0.0001
Suicidal ideation (QIDS-SR item 12)	No (0)	500 (73)	106 (54.4)	<0.0001
	Yes (>1)	185 (27)	89 (45.6)	
YMRS	0	370 (54)	92 (47.2)	0.24
	(1–7)	250 (36.5)	81 (41.5)	
	>7	65 (9.5)	22 (11.3)	
PSQI (0–21)		6.52 (3.58)	9.021 (4.15)	<0.0001
STAI-Y (state) (0–60)		40.88 (14.01)	48.35 (14.18)	<0.0001
MAThyS Emotional (0–40)		20.99 (6.49)	22.93 (7.12)	0.0003
MAThyS Motivation (0–40)		17.56 (6.65)	16.07 (8.07)	0.009
MAThyS Cognition (0–40)		20.31 (5.85)	20.72 (7.05)	0.4
MAThyS Sensory perception (0–50)		25.89 (4.58)	25.81 (7.16)	0.86
MAThyS Psychomotor (0–30)		12.83 (5.36)	11.90 (6.22)	0.04
AIM		3.66 (0.69)	3.93 (0.63)	<0.0001
ALS		1.18 (0.67)	1.55 (0.63)	<0.0001
BDHI Expressive Component		20.07 (7.90)	23.15(8.133)	0.0001
BDHI Attitudinal Component		6.99 (4.28)	8.97 (4.20)	<0.0001
BIS-10		66.19 (11.12)	70.51 (11.57)	<0.0001
Lithium carbonate	No	425 (62)	143 (73.3)	0.005
	Yes	260 (38)	52 (26.7)	
Anticonvulsant	No	340 (49.6)	84 (43.1)	0.12
	Yes	345 (50.4)	111 (56.9)	
Antipsychotic	No	369 (53.9)	108 (55.4)	0.77
	Yes	316 (46.1)	87 (44.6)	
Anxiolytic	No	521 (76.1)	136 (69.7)	0.09
	Yes	164 (23.9)	59 (30.3)	
Hypnotic	No	580 (84.7)	157 (80.5)	0.2
	Yes	105 (15.3)	38 (19.5)	
Antidepressant	No	420 (61.3)	107 (54.9)	0.12
	Yes	265 (38.7)	88 (45.1)	

QIDS-SR Quick Inventory of Depressive Self-report; YMRS Young Mania Rating Scale; PSQI Pittsburgh Sleep Quality Index; STAI-Y State–Trait Anxiety Inventory; MAThyS Multidimensional Assessment of Thymic States; AIM Affect Intensity Measure; ALS Affective Lability Scale; BDHI Buss–Durkee Hostility Inventory; BIS-10 Barratt Impulsiveness Scale.

**Table 2:** Odds ratios for each best model selected based on the AIC.

	Model 1	Model 2	Model 3
Age	1.01 (1.00–1.03)	1.01 (1.00–1.03)	1.02 (1.00–1.03)
Education (high school diploma)	0.79 (0.56–1.13)	0.78 (0.54–1.11)	0.87 (0.60–1.26)
PSQI	1.10 (1.04–1.15)	1.09 (1.04–1.15)	1.08 (1.03–1.14)
QIDS-SR (without item 12) Box–Cox	1.19 (1.09–1.30)	1.19 (1.09–1.30)	1.14 (1.04–1.25)
MAThyS emotional ☨			
MAThyS sensory ☨			
BD subtype II ✦		1.11 (0.76–1.62)	
BD subtype NOS ✦		0.78 (0.42–1.45)	
“Borderline personality traits” ✧			1.13 (1.00–1.29)

PSQI Pittsburgh Sleep Quality Index; QIDS-SR Quick Inventory of Depressive Self-report; MAThyS Multidimensional Assessment of Thymic States; BD Bipolar disorder. ☨ The sensory and emotional subcomponents of the MAThyS were included in the three models, but the odds ratios could not be extracted for these two variables because their relationship with pain was quadratic. ✦ The reference for comparison is BD subtype I. ✧ “Borderline personality traits” are defined by the combination of affective lability, affective intensity, hostility/anger, and impulsivity.

**Table 3:** Correlation between personality trait questionnaire scores and the first PCA component (i.e., “borderline personality traits”).

Questionnaires	Correlation with the First PCA Component: “Borderline Personality Traits” ✧
AIM	0.77
ALS	0.87
BDHI Attitudinal Component	0.76
BDHI Expressive Component	0.78
BIS-10	0.70

AIM Affect Intensity Measure; ALS Affective Lability Scale; BDHI Buss–Durkee Hostility Inventory; BIS-10 Barratt Impulsiveness Scale. ✧ “Borderline personality traits” are defined by the combination of affective lability, affective intensity, hostility/anger, and impulsivity.

## Data Availability

The datasets generated and/or analyzed during the current study are not publicly available due to the sensitive and identifiable nature of health data but are available from the corresponding author on reasonable request.

## Supplementary Materials

The following are available online at https://www.mdpi.com/article/10.3390/jcm11030893/s1, Table S1: Sociodemographic and clinical characteristics of patients lost and not lost to follow-up. Table S2. Eigenvalue and variance explained by each dimension of the PCA.
