# Supplementary material for: Self-Reported Pain and Emotional Reactivity in Bipolar Disorder: A Prospective FACE-BD Study
Source: J Clin Med. 2022 Feb 8;11(3):893. doi: 10.3390/jcm11030893 (PMC8836480; doi:10.3390/jcm11030893)
Supplement: Supplementary file 1 [file jcm-11-00893-s001.zip › Table_S1.pdf]

Table S1: Sociodemographic and clinical characteristics of patients lost and not lost to follow-up.

| Variable | | Patients lost to follow-up Mean (sd)/ Number (%) | Patients not lost to follow-up Mean (sd)/ Number (%) | P -value |
|---|---|---|---|---|
| **Sociodemographic** | | | | |
| Age (years) | | 38.85 (12.23) | 42.58 (12.94) | <0.0001 |
| Sex | Men | 207(38.6) | 141(41) | 0.53 |
| | Women | 329(61.4) | 203(59) | |
| Single | No | 266 (49.6) | 187 (54.4) | 0.19 |
| | Yes | 270 (50.4) | 157 (45.6) | |
| Education (High school diploma) | No | 186(34.7) | 144(41.9) | 0.04 |
| | Yes | 350(65.3) | 200(58.1) | |
| **Clinical** | | | | |
| BD subtype | I | 246(45.9) | 163(47.4) | 0.17 |
| | II | 227(42.4) | 154(44.8) | |
| | NOS | 63(11.8) | 27(7.8) | |
| Age at BD onset (years) | | 23.082(8.928) | 24.347(9.556) | 0.05 |
| Number of depressive episodes | | 5.234(4.947) | 5.366(4.605) | 0.72 |
| Number of manic episodes | | 1.074(2.146) | 1.074(2.07) | 1 |
| Number of hypomanic episodes | | 3.821(5.471) | 2.985(4.075) | 0.03 |
| Lifetime history of suicide attempt | No | 364(67.9) | 220(64) | 0.25 |
| | Yes | 172(32.1) | 124(36) | |
| Current substance use disorder | No | 469(87.5) | 314(91.3) | 0.1 |
| | Yes | 67(12.5) | 30(8.7) | |
| Lifetime anxiety disorder | No | 311(58) | 220(64) | 0.09 |
| | Yes | 225(42) | 124(36) | |
| Lifetime eating disorder | No | 424(79.1) | 286(83.1) | 0.16 |
| | Yes | 112(20.9) | 58(16.9) | |
| Multiple sclerosis | No | 515(99.6) | 334(99.4) | 0.65 |
| | Yes | 2(0.4) | 2(0.6) | |
| Cancer | No | 481(96.8) | 322(97.6) | 0.65 |
| | Yes | 16(3.2) | 8(2.4) | |
| Inflammatory bowel disease | No | 513(99.4) | 330(98.8) | 0.44 |
| | Yes | 3(0.6) | 4(1.2) | |
| Rheumatoid arthritis | No | 523(99.8) | 338(100) | 1 |

| | | | | |
|---|---|---|---|---|
| | Yes | 1(0.2) | 0(0) | |
| Ulcer | No | 498(96.7) | 322(96.7) | 1 |
| | Yes | 17(3.3) | 11(3.3) | |
| QIDS-SR (without item 12) Box Cox Transformed | | 5.321(2.579) | 5.224(2.547) | 0.59 |
| Suicidal ideation (QIDS-SR item 12) | No (0) | 372(69.4) | 234(68) | 0.72 |
| | Yes (>1) | 164(30.6) | 110(32) | |
| YMRS | 0 | 269(50.2) | 193(56.1) | 0.12 |
| | [1-7] | 216(40.3) | 115(33.4) | |
| | > 7 | 51(9.5) | 36(10.5) | |
| PSQI [0-21] | | 7.159(3.899) | 6.959(3.799) | 0.46 |
| STAI-Y (state) [0-60] | | 42.771(14.583) | 42.177(14.087) | 0.55 |
| MAThyS Emotional [0-40] | | 21.266(6.684) | 21.675(6.681) | 0.38 |
| MAThyS Motivation [0-40] | | 17.195(7.083) | 17.288(6.932) | 0.85 |
| MAThyS Cognition [0-40] | | 20.357(6.132) | 20.482(6.147) | 0.77 |
| MAThyS Sensory perception [0-50] | | 25.605(5.454) | 26.296(4.928) | 0.06 |
| MAThyS Psychomotor [0-30] | | 12.7(5.736) | 12.515(5.333) | 0.63 |
| AIM | | 3.724(0.678) | 3.707(0.701) | 0.72 |
| ALS | | 1.271(0.684) | 1.249(0.687) | 0.65 |
| BDHI Expressive Component | | 21.192(8.218) | 20.078(7.751) | 0.045 |
| BDHI Attitudinal Component | | 7.563(4.404) | 7.23(4.244) | 0.27 |
| BIS-10 | | 67.377(11.522) | 66.805(11.121) | 0.47 |
| Lithium carbonate | No | 366(68.3) | 202(58.7) | 0.005 |
| | Yes | 170(31.7) | 142(41.3) | |
| Anticonvulsant | No | 295(55) | 129(37.5) | <0.0001 |
| | Yes | 241(45) | 215(62.5) | |
| Antipsychotic | No | 320(59.7) | 157(45.6) | 0.0001 |
| | Yes | 216(40.3) | 187(54.4) | |
| Anxiolytic | No | 424(79.1) | 233(67.7) | 0.0002 |
| | Yes | 112(20.9) | 111(32.3) | |
| Hypnotic | No | 468(87.3) | 269(78.2) | 0.0005 |
| | Yes | 68(12.7) | 75(21.8) | |
| Antidepressant | No | 356(66.4) | 171(49.7) | <0.0001 |
| | Yes | 180(33.6) | 173(50.3) | |

**QIDS-SR** Quick Inventory of Depressive Self-report; **YMRS** Young Mania Rating Scale; **PSQI** Pittsburgh Sleep Quality Index; **STAI-Y** State-Trait Anxiety Inventory; **MAThyS** Multidimensional Assessment of Thymic States; **AIM** Affect Intensity Measure; **ALS** Affective Lability Scale; **BDHI** Buss-Durkee Hostility Inventory; **BIS-10** Barratt Impulsiveness Scale
