# Supplementary material for: Self-Reported Pain and Emotional Reactivity in Bipolar Disorder: A Prospective FACE-BD Study
Source: J Clin Med. 2022 Feb 8;11(3):893. doi: 10.3390/jcm11030893 (PMC8836480; doi:10.3390/jcm11030893)
Supplement: Supplementary file 1 [file jcm-11-00893-s001.zip › Table_S2.pdf]

Table S2: Eigenvalue and variance explained by each dimension of the PCA.

PCA Dimension	Eigenvalue	Percentage of variance explained by the dimension	Cumulative percentage of variance
Dimension 1	3.025	60.493	60.493
Dimension 2	0.654	13.085	73.578
Dimension 3	0.577	11.534	85.112
Dimension 4	0.444	8.873	93.985
Dimension 5	0.301	6.015	100.00
